# Genome data on the extinct *Bison schoetensacki* establish it as a sister species of the extant European bison (*Bison bonasus*)

**DOI:** 10.1186/s12862-017-0894-2

**Published:** 2017-02-10

**Authors:** Pauline Palacio, Véronique Berthonaud, Claude Guérin, Josie Lambourdière, Frédéric Maksud, Michel Philippe, Delphine Plaire, Thomas Stafford, Marie-Claude Marsolier-Kergoat, Jean-Marc Elalouf

**Affiliations:** 1 Institute for Integrative Biology of the Cell (I2BC), IBITECS, CEA, CNRS, Université Paris-Sud, Université Paris-Saclay, 91198 Gif-sur-Yvette cedex, France; 2 CNRS-UMR 5276, Laboratoire de Géologie de Lyon: Terre, planètes, environnement, Département des Sciences de la Terre, Université Claude Bernard, Lyon I, 27-43 Boulevard du 11 Novembre, 69622 Villeurbanne cedex, France; 3 Service de Systématique Moléculaire, UMS 2700 CNRS-MNHN, CP26, 57 Rue Cuvier, 75231 Paris Cedex 05, France; 4 Service Régional de l’Archéologie, 32 rue de la Dalbade, BP811 31080 Toulouse cedex 6, France; 5 Centre de Conservation et d’Étude sur les Collections, 13A rue Bancel, 69007 Lyon, France; 6 CNRS-UMR 7206, Eco-anthropologie et Ethnobiologie, Département Hommes, Natures et Sociétés, Musée de l’Homme, 17 place du Trocadéro et du 11 novembre, 75016 Paris, France; 7 Stafford Research, 200 Acadia Avenue, Lafayette, CO 80026 USA

**Keywords:** Ancient DNA, Bovinae, Cave hyena, Coprolite, *Crocuta crocuta*, Mitochondrial genome, Paleogenomics

## Abstract

**Background:**

The European bison (*Bison bonasus*), now found in Europe and the Caucasus, has been proposed to originate either from the extinct steppe/extant American bison lineage or from the extinct *Bison schoetensacki* lineage. *Bison schoetensacki* remains are documented in Eurasian Middle Pleistocene sites, but their presence in Upper Pleistocene sites has been questioned. Despite extensive genetic studies carried out on the steppe and European bison, no remains from the fossil record morphologically identified as *Bison schoetensacki* has been analyzed up to now.

**Results:**

In this paper, we analyzed a 36,000-year-old *Bison schoetensaki* bone sample from the Siréjol cave (France) and a cave hyena coprolite (fossilized feces) found in a nearby cave and containing large amounts of Bovinae DNA. We show that the Bovinae mitochondrial DNA sequences from both samples, including a complete mitochondrial genome sequence, belong to a clade recently reported in the literature. This clade only includes ancient bison specimens without taxonomic identification and displays a sister relationship with the extant European bison. The genetic proximity of *Bison schoetensacki* with specimens from this clade is corroborated by the analysis of nuclear DNA single nucleotide polymorphisms.

**Conclusions:**

This work provides genetic evidence supporting the continuing presence of *Bison schoetensacki* up to the Upper Pleistocene. *Bison schoetensacki* turns out to be a sister species of *Bison bonasus*, excluding the steppe bison *Bison priscus* as a direct ancestor of the European bison.

**Electronic supplementary material:**

The online version of this article (doi:10.1186/s12862-017-0894-2) contains supplementary material, which is available to authorized users.

## Background

During the Middle and Upper Pleistocene, the large Bovidae in Europe and in northern Asia chiefly included the aurochs, *Bos primigenius* (Bojanus, 1827), and two bison species, the steppe bison and the woodland bison. The steppe bison, *Bison priscus* (Bojanus, 1827), was very common and exhibited a wide geographic distribution stretching from western Europe, through Central Asia and Beringia, and into North America. It was a formidable animal with long horns and robust legs; it stood up to two meters at the withers and reached a total length of almost three meters. It occupied cool, steppe-like grasslands. *Bison priscus* became extinct in Europe at the end of the last Ice Age, about 12,000 years ago [1].

**Fig. 1:**
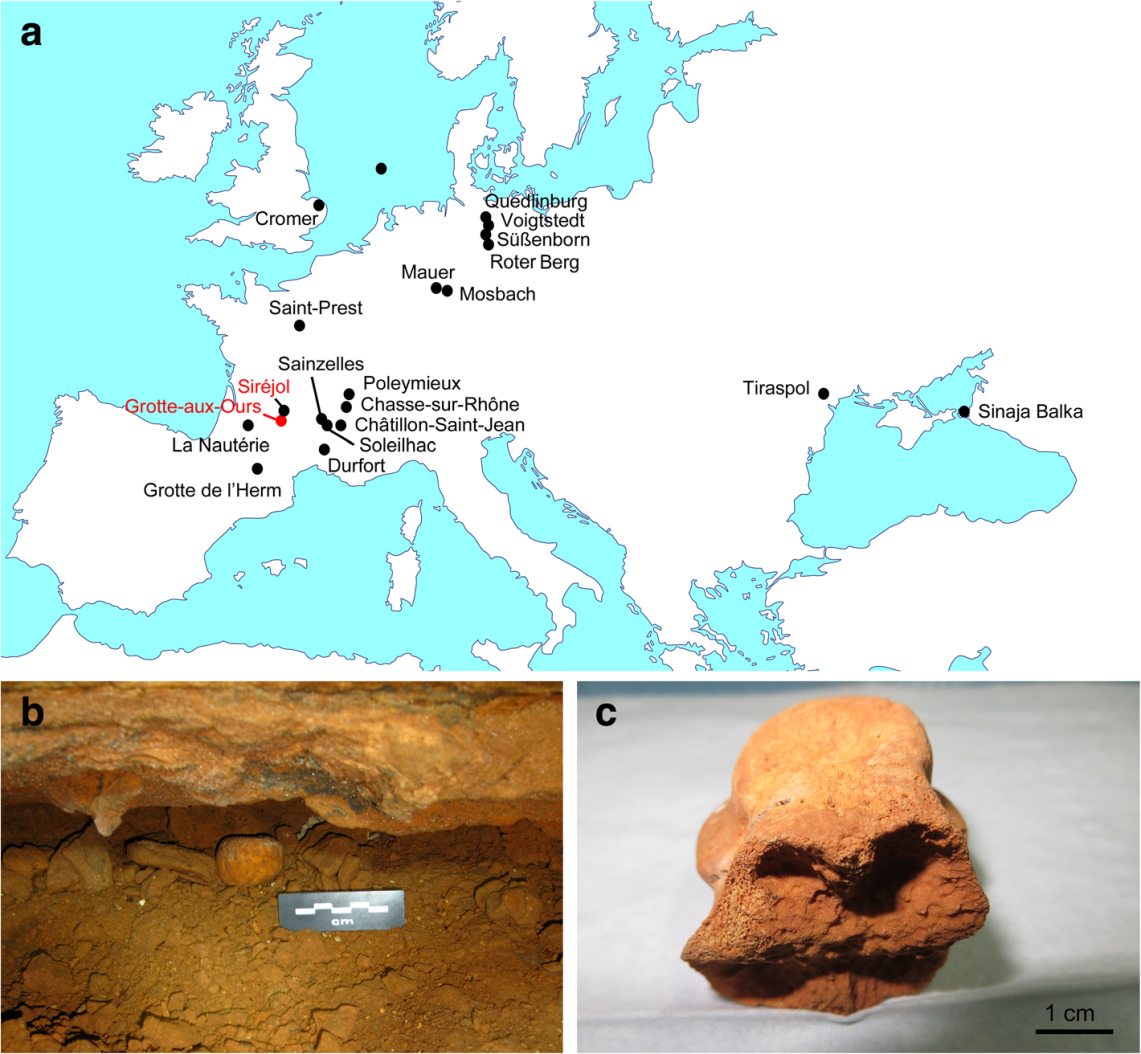
Cave sites and samples. (**a**) Black dots indicate sites where *Bison schoetensacki* remains have been reported in previous studies. Red characters indicate the two cave sites that yielded the samples analyzed in the present study. (**b**) The coprolite from the Grotte-aux-Ours cave (GAO1 sample). (**c**) The *Bison schoetensacki* cannon bone sample from the Siréjol cave (Siréjol 20101699 sample)

The woodland bison, *Bison schoetensacki* (Freudenberg, 1910) appeared in the early Middle Pleistocene. Its size was almost as large as that of *Bison priscus* but its leg bones and metapodials were slenderer than those of *Bison priscus* [2, 3]. Moreover the horns of *Bison schoetensacki* were about 30% shorter than those of *Bison priscus* and displayed a slightly different shape: the initial third part is oriented forward, the second third goes upward, and the final part slightly bends backward. *Bison schoetensacki* remains are often associated with forest biotopes, hence its vernacular name. The geographic distribution of *Bison schoetensacki* extended from western Europe to the south of Siberia, but unlike *Bison priscus*, *Bison schoetensacki* was absent from Beringia and North America. The species was first described in Germany where it has been recorded in several lower Middle Pleistocene sites, notably in Mosbach, Mauer, Süßenborn and Voigtstedt, but also in Roter Berg and Quedlinburg (Fig. 1a). It has also been found in England (Cromer Forest Bed), in Moldova (Tiraspol), in Russia (Sinaja Balka), and in North Sea deposits. In France, *Bison schoetensacki* is recorded in several lower Middle Pleistocene sites (Durfort [4], Saint-Prest, La Nautérie, Soleilhac, Sainzelles) and in late Middle Pleistocene deposits in Châtillon-Saint-Jean [5]. It seems to be still present during the Upper Pleistocène at Chasse-sur-Rhône, Poleymieux and in the Herm and Siréjol caves. However the persistence of *Bison schoetensacki* during the upper Pleistocene is under debate, and Siréjol bone remains have been alternatively ascribed to *Bison schoetensacki* [6] and to a *Bison priscus* novel subspecies [7]. *Bison schoetensacki* remains from Siréjol display a cranial frontal no larger than 214 mm, short horns of the shape described above with maximal vertical and anteroposterior diameter at their base of 63 and 65 mm, respectively [6].

Two bison species exist today: the American bison and the European bison. The genetic history of the American bison (*Bison bison*) has been extensively investigated through the analysis of mitochondrial DNA sequences [8–11]. The recent reconstruction of the complete mitochondrial genome of a 19,000-year-old *Bison priscus* specimen confirmed that the closest extant mitochondrial genome sequences for *Bison priscus* are those of *Bison bison* [12]. These data, in combination with nuclear single nucleotide polymorphism (SNP) genotyping [13], have established that *Bison priscus* and *Bison bison* are sister groups.

The European bison, *Bison bonasus,* is found in Europe and the Caucasus, where it has been reintroduced after its extinction as a wild species in the early 20^th^ century. The *Bison bonasus* genomes reflect a complex descent. Indeed, the *Bison bonasus* nuclear genome is closely related to that of *Bison bison* [14–16], in agreement with morphological evidence and the fact that the two bison species can produce completely fertile hybrid offspring. However, the mitochondrial genomes of *Bison bonasus* specimens are more similar to aurochs and cattle genomes than to *Bison bison* genomes [14, 17, 18]. These observations could be explained either by incomplete lineage sorting of the mitochondrial genome or by a scenario according to which the nuclear DNA of an ancient population probably related to the extinct aurochs (itself closely related to cattle) would have been changed by the systematic introgression of bison bulls [10, 14, 18]. The phylogenetic relationships between *Bison bonasus*, *Bison priscus* and *Bison schoetensacki* are subject to debate. Some authors suggest that *Bison schoetensacki* could be the ancestor of *Bison bonasus* [1], but others consider that *Bison bonasus* is derived from an unknown form of *Bison priscus* [19, 20]. The lack of genomic data for *Bison schoetensacki* has so far prevented any conclusive view on this point.

As part of an effort to obtain genetic data on animals consumed by the extinct cave hyena (*Crocuta crocuta*), we initiated the analysis of cave hyena coprolites [21]. In the present study, we found a coprolite containing mitochondrial Bovinae DNA that was different from *Bos primigenius*, *Bison priscus* and *Bison bonasus* mitochondrial sequences. Because the cave site (Grotte-aux-Ours, Fig. 1a) that yielded this coprolite is located close to the Siréjol cave, we also analyzed DNA extracted from a Siréjol *Bison. schoetensacki* bone sample. We report here on the mitochondrial DNA sequences (including a complete mitochondrial genome sequence) obtained from the coprolite and the bone remain, on their phylogenetic positions, and on a set of SNPs of the corresponding nuclear genome sequence.

## Methods

### Archeological samples

The cave hyena coprolite was retrieved from the Grotte-aux-Ours cave (Souillac, Lot, France). The cave was discovered in 2008 and owes its name to the abundant evidence for long-term occupation by the extinct cave bear (*Ursus spelaeus*), including cave bear bones, hibernation nests, claw marks on the walls, and footprints on the paleosurface. Occupation by the extinct cave hyena (*Crocuta crocuta*) is documented by the presence of coprolites and a cave hyena skull.

The *Bison schoetensacki* bone fragment originated from the Siréjol cave (Gignac, Lot, France). This cave, discovered in 1964, is located 15 km away from the Grotte-aux-Ours cave. Scientific excavations carried out from 1972 to 1975 in the Siréjol cave yielded *Bison schoetensacki* bone remains [22] that are currently stored in the Centre de Conservation et d’Étude des collections du Musée des Confluences (Lyon, France). For the purpose of the present study, we analyzed a *Bison schoetensacki* cannon bone fragment (registration number: 20101699).

### Contamination issues

To guarantee that genuine ancient DNA was recovered from the samples, DNA extraction was carried out from material derived from the inside of the samples, and the coprolite and bone DNA extracts were obtained on different days. Additional precautions to avoid contamination have been described elsewhere [12, 21]. Negative controls included mock extracts and PCR blanks (where water was added instead of DNA extracts), which always failed to yield any amplification product.

### DNA extraction

The instruments used to cut and pulverize the samples were cleaned in an ultrasonic water-tank, incubated 1 h at 40 °C in 0.1% SDS, rinsed 4 times in ultrapure DNase/RNase free distilled water, and dried using absolute ethanol. The coprolite and the bone sample were cut using a carbon-free circular saw. For DNA extraction we used 0.5 g of biological material retrieved from the center of the coprolite or the bone medulla using a sterile single-use scalpel. The dissected material was pulverized using a Mikro-Dismembrator S (Sartorius; Goettingen, Germany) set to a shaking frequency of 2,000 rpm for 30 s, transferred into 6 ml of DNA extraction buffer (0.45 M EDTA, 10 mM Tris-HCl (pH 8.0), 0.1% SDS, 65 mM DTT, 0.5 mg/ml proteinase K), and incubated 16 h at 42 °C under constant agitation. After centrifugation, the supernatant was recovered, extracted once with one volume of phenol, once with a phenol-chloroform (50:50) mixture, and once with chloroform. The aqueous phase was dialyzed and concentrated using a 2 ml, centrifugal ultrafilter with a 30 kDa cutoff (Millipore, Billerica, MA), the column was washed 4 times with distilled water, and the DNA extract was recovered in a volume of 100 to 120 μl. It was purified further using a silica-membrane-based method designed for small DNA fragments (Qiagen purification kit #28004; Venlo, Netherlands). The final extract had a volume of 100 μl.

### PCR and Sanger sequencing DNA analysis

PCR reactions were performed in a 50-μl reaction volume containing 0.2–0.4 μl of mock or ancient DNA extracts, 300 nM of sense and antisense primers, 200 μM dNTP, 2.5 mM MgCl_2_, 5 μl of GeneAmp 10X PCR buffer II, and 2.5 U of AmpliTaq Gold DNA polymerase (Thermo Fisher Scientific, Waltham, MA, USA). The primers used for experiments designed to complete the bovine mitochondrial genome sequence assembled from the Grotte-aux-Ours coprolite are listed in Additional file 1: Table S1. The primers used to analyze mitochondrial genome fragments of *Bison schoetensacki* bone sample from the Siréjol cave are listed in Additional file 1: Table S2. Primer pair 12 from Additional file 1: Table S2 was used for initial screening of the samples for Bovinae DNA content. PCR procedures consisted of an enzyme activation step (95 °C, 8.5 min), followed by a single round of 45 PCR cycles (95 °C, 15 s; 46–58 °C (according to primers *T*_*m*_), 20 s; 70 °C, 1 min) performed in a Veriti thermal cycler (Thermo Fisher Scientific).

The full reaction volume was loaded onto an 8% polyacrylamide gel stained with Sybr Green I (Thermo Fisher Scientific). Only the coprolite and bone extracts yielded amplification products of the predicted size. PCR amplicons were eluted from the gel and cloned using the TOPO TA cloning kit (Thermo Fisher Scientific). Sequencing analysis of cloned DNA fragments was performed on ABI 3130 or 3130XL automatic DNA sequencers using M13 forward primer and BigDye 3.1 terminator chemistry (Thermo Fisher Scientific). All mitochondrial genome fragments analyzed by PCR were characterized through 2–6 PCR replicates and a consensus sequence for each replicate was derived from several clones (average number of clones, 9; range, 3–26). For the bovine mitochondrial genome sequence reconstructed from the Grotte-aux-Ours sample, each individual consensus was added to the Illumina sequence data (see below). For the Siréjol bone sample, several individual consensuses of the same fragments were combined to deduce the most relevant base of the 609 positions of the *Bison schoetensacki* mitochondrial genome sequence investigated by PCR.

### Generation and sequencing of a library of DNA fragments

A library of DNA fragments suitable for high-throughput sequencing with the Illumina procedure [23] was generated from the coprolite DNA extract using the Illumina TruSeq Nano DNA LT sample kit FC-121-4001. We followed the manufacturer’s recommendations (San Diego, CA, USA), except for modifications that were introduced for the purpose of analyzing ancient, *i.e.* highly fragmented DNA fragments. Thus, the initial step (DNA shearing) of the library construction process was omitted, and reaction products were purified using Qiagen columns or polyacrylamide gel electrophoresis instead of magnetic beads. The process of library construction consisted of 5 steps. First, 6 μl of coprolite DNA was 5’end-phosphorylated and blunt-ended using a mixture containing T4 polynucleotide kinase, T4 and Klenow DNA polymerases, and the reaction product was purified on a Qiagen 28004 column. Second, a 3’ adenine residue was added to the blunt-ended DNA fragments using Klenow 3’ to 5’ exo- polymerase, and the enzyme was heat-inactivated at 70 °C. Third, Illumina adapters with an overhanging thymine were ligated to the DNA fragments; the reaction product was purified using a Qiagen 28104 column and recovered in a volume of 30 μl referred to as the DNA library. Fourth, a 5-μl aliquot of the library was PCR-amplified using Phusion DNA polymerase (95 °C, 3 min for enzyme activation, followed by 12 PCR cycles of 98 °C for 20s, 60 °C for 15 s, and 72 °C for 30s). Fifth, the full PCR reaction volume was loaded on an 8% polyacrylamide gel stained with SYBR Green I, and the 150 to 220-bp long DNA fragments (consisting of 122-bp derived from the adapters, and about 30 to 100-bp derived from the sample) were cut off the gel and purified.

DNA sequencing was performed at Genoscope (Evry, France) on the Illumina HiSeq 2500 platform (4 lines of an 8-line flow-cell) using HiSEQ v3 chemistry with a read length set to 101 nucleotides and analysis on the single read mode.

### Illumina sequencing data analysis

Reads were trimmed for adapter sequences, N’s and low quality stretches on the 3’ end, using a software based on the FASTX-Toolkit package (http://hannonlab.cshl.edu/fastx_toolkit) and designed by Genoscope. After this step, sequences shorter than 20 nucleotides were discarded using an in-house Python script, yielding a dataset of 601,509,879 DNA reads.

### Initial analysis of the Illumina library content

To confirm the identity of the coprolite producer and gain insight into the animal’s diet, the 601,509,879 Illumina reads were aligned simultaneously to a set of 49 mitochondrial genomes, including the reference mitochondrial genomes for the extinct cave hyena (*Crocuta crocuta*, GenBank accession number NC_020670.1) and for several potential prey species (Additional file 1: Table S3). The alignment was performed using BWA version 0.7.12 [24]. Only the reads matching perfectly, without indel or mismatch, to a unique genome were taken into account.

### Assembling simultaneously the bovine and the cave hyena mitochondrial genomes

The sequence assembly was performed in several steps. First the Illumina reads, ranging in size from 20 to 101 nucleotides, were aligned simultaneously to the reference mitochondrial genomes of the extant European bison (*Bison bonasus*, GenBank accession number NC_014044.1) and extinct cave hyena (*Crocuta crocuta*) using BWA version 0.7.12 [24] with default options except for *-o* (gap opening) and *-n* (maximum edit distance), which were set to 1 and 0.1, respectively, allowing for a maximum edit distance that increases with read length, from one mismatch for 20–26 nucleotide-long reads to four mismatches for 88–101 nucleotide-long reads. In this first round, the number of reads mapping to the *Bison bonasus* genome (20,384) was of the same order of magnitude as the number of reads mapping to the *Crocuta crocuta* genome (13,318). Of significance, 1,201 reads mapped to both genomes, which justified our strategy to simultaneously assemble the two mitochondrial genomes so as to eliminate spurious alignments. A set of 17,669 unique reads mapping exclusively on the *Bison bonasus* genome (and not on the *Crocuta crocuta* genome) with a mapping quality higher than 25 was selected. The reads provided a median coverage of 28 of the *Bison bonasus* reference sequence and left 254 positions without coverage. A provisional consensus sequence termed BB1seq was then derived from the 17,669 unique reads. BB1seq differed from the *Bison bonasus* reference genome at 289 positions. In this study a consensus for a given position was based on at least two concordant unique sequences (either Illumina reads or consensus PCR sequences) with a minimal mapping quality of 25 for Illumina reads. The most frequent base was taken as the consensus. The few cases where more than 25% of the bases from the aligned reads differed from the consensus were manually examined: the large majority of the alternative bases could be ascribed to damage-induced G to A substitutions at the 3' end of the Illumina reads and the other cases were checked by sequence data obtained from PCR studies.

The cave hyena mitochondrial genome was assembled in parallel, using the same procedure. A provisional consensus sequence termed CC1seq was derived from 12,139 unique reads that aligned specifically to the *Crocuta crocuta* reference mitochondrial genome. The reads gave a median coverage of 27 for the *Crocuta crocuta* reference sequence and left 380 positions uncovered. CC1seq and the *Crocuta crocuta* reference genome differed at 29 positions.

In a second step, all Illumina reads were realigned simultaneously to the provisional consensus sequences BB1seq and CC1seq and new consensus sequences, termed BB2seq for the bovine genome and CC2seq for the cave hyena genome, were derived following the procedure previously described. For the bovine sequence, PCR experiments were then carried out to fill in the gaps (169 positions) and derive a robust sequence at positions where only a single read was available (144 positions). The 27 PCR primer pairs were designed using the BB2seq sequence (see above PCR and Sanger sequencing DNA analysis). A total of 84 consensus PCR sequences were used to complement BB2seq information and to derive a new consensus sequence, termed BB3seq. All Illumina reads were again simultaneously aligned to CC2seq and BB3seq. After a last iteration, we were able to establish the final, complete consensus sequence, termed GAOseq_Bovinae, for the bovine mitochondrial genome, based on 19,830 unique Illumina reads, and a partial sequence for the cave hyena mitochondrial genome, called GAOseq_Crocuta, based on 12,338 unique Illumina reads.

### Annotation of the GAOseq_Bovinae mitochondrial genome

The annotation of GAOseq_Bovinae was derived from the annotation of the *Bison bonasus* reference mitochondrial genome. Briefly, GAOseq_Bovinae and the *Bison bonasus* reference mitochondrial genome were aligned using the LAGAN software [25] with the annotation data of the *Bison bonasus* genome (http://www.ncbi.nlm.nih.gov/nuccore/ NC_014044.1). LAGAN produced a list of the aligned genomic features with their coordinates in the two genomes, which was manually checked. The occurrence of a start and stop codon at the beginning and the end of each coding sequence, and the absence of internal stop codons were verified using an in-house Python script.

### Phylogenetic analyses

All phylogenetic analyses were performed using MEGA [26]. The phylogenetic relationships between GAOseq_Bovinae and the complete mitochondrial genomes of specimens related to *Bison bonasus* were inferred using the Maximum Likelihood (ML), the Minimum Evolution and the Neighbor-Joining methods. The ML method was based on the General Time Reversible model. A discrete Gamma distribution was used to model evolutionary rate differences among sites (5 categories). The analysis involved a total of 15,332 positions.

A similar approach was used to analyze the DNA sequences of the *Bison schoetensacki* bone sample. Sixteen consensus sequences, each one derived from several clones of several PCR replicates, were aligned to the set of bison sequences previously used for the analysis of the complete mitochondrial genomes. The aligned parts were subsequently concatenated and a phylogenetic tree was constructed using the ML method based on the Tamura-Nei model [27] and a discrete Gamma distribution to model evolutionary rate differences among sites (5 categories).

### Analysis of the bovine nuclear sequences

In order to compare the bison nuclear sequences of the coprolite to the genomic data (~10,000 genome-wide bovine SNPs) made recently available for ancient specimens related to *Bison bonasus* [28], the library reads were mapped against the taurine cattle reference UMD 3.1 [29] using BWA, and the bovine SNPs were called using SAMtools and BCFtools v1.2 [30]. The calls were filtered using the same criteria as in [28] (QUAL value higher than 25, minimum depth of coverage at 2 for the variants). The final number of SNPs called for the bovine sequences of the coprolite was 1,819, which is comparable to the number of SNPs called for the A4093 sample (ancient wisent, 1,946 SNPs), but lower than the number of SNPs called for the other bison samples (5,690 SNPs for the CladeX sample A006 and > 8,400 for the other bison samples).

These data were completed by extracting the sequences of 7,921 SNPs from the set of 22,129 unplaced genomic scaffolds corresponding to the nuclear genome of *Bison bison bison* isolate TAMUID 2011002044 (retrieved from the NCBI website http://www.ncbi.nlm.nih.gov/nuccore?term=257088[BioProject] on June, 23rd 2015).

Multidimensional scaling analysis was performed using the R software v3.0.1 [31] and in particular the cmdscale() function.

### AMS ^14^C dating

Radiocarbon dating was attempted on both the hyena coprolite and the *Bison schoetensacki* bone sample. Only the bone sample yielded AMS ^14^C measurements. No bone, hair, or macrofloral remains were present in the coprolite, and what constituted the matrix dissolved during weak-alkali (0.02 M KOH) extraction. Consequently, no direct ^14^C measurements were possible on the coprolite.

Part of the Siréjol bone sample was broken into 3–4 mm fragments, which were washed in deionized (DI) water with brief sonication to remove adhering sediment. The bone fragments were subsequently decalcified with repeated changes of 0.2 N HCl for 2 days at 4 °C and the acid-insoluble collagen washed to neutrality with DI water. The collagen was treated with 0.1 M KOH overnight at 4 °C to remove humic acids. An aliquot of the alkali-extracted fraction was washed to neutrality with DI water, acidified with 0.05 N HCl, freeze dried and used for ^14^C dating of the KOH-extracted, decalcified collagen fraction (D-AMS 012204).

The remainder of the KOH-extracted collagen was used for gelatin extraction, which comprised heating the alkali-extracted collagen to 90 °C in 0.05 N HCl until dissolution occurred, in 45 min. The gelatin solution was filtered through a 0.45 μm Durapore filter membrane and freeze dried. Approximately 5 mg of collagen and gelatin were weighed into quartz tubes with CuO and Ag, the tubes evacuated to < 10 mTorr under LN pumping, sealed with a H_2_/O_2_ torch and the evacuated tubes combusted at 850 °C for 1 h. The CO_2_ was isolated cryogenically, converted into graphite using the H_2_-Fe method [32] and used for ^14^C dating of the gelatin fraction (D-AMS 012205).

Both fractions were dated at the Direct AMS Accelerator Facility, Seattle, Washington USA. The calibrated age of the sample, expressed in calendar years before present (cal yr BP), was calculated using OxCal 4.2 software [33] and the IntCal 13 calibration curve [34].

## Results and Discussion

### Analysis of the coprolite collected from the Grotte-aux-Ours cave

**Fig. 2:**
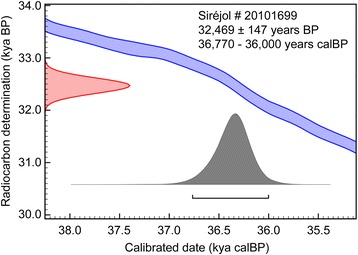
Uncalibrated (BP) and calibrated (calBP) age of the Siréjol *Bison schoetensacki* bone sample. The calBP data correspond to 95.4% (2 σ) confidence interval of the sample age using IntCal13 calibration curve

Figure 1b-c shows the coprolite collected in the Grotte-aux-Ours cave and the *Bison schoetensacki* bone fragment from the Siréjol cave. We obtained for the bone fragment radiocarbon dates of 32,316 ± 215 and 32,623 ± 200 BP using the KOH-extracted decalcified collagen and the gelatin fraction of the sample, respectively. We considered the average value (32,469 ± 147 BP) of these two measurements as the most reliable determination of the sample age, which corresponds to 36,770–36,000 cal yr BP (Fig. 2, Additional file 1: Table S4). Previous analyses performed using conventional ^14^C dating of bulk bone material from the same cave sector yielded ages of 31,300 [+1,800/-1,600] BP and 29,100 [+1,600/-1,300] BP [35]. Our AMS radiocarbon studies agree with this time frame and greatly increase the accuracy of dating analysis of the Siréjol material. Dating analysis of the coprolite failed because the sample completely dissolved during the pretreatment for AMS measurement.

PCR analysis of the coprolite DNA for the mitochondrial cytB fragments demonstrated the presence of cave hyena DNA and of a Bovinae sequence different from all of those recorded in GenBank up to March 2016. The 82-bp Bovinae cytB fragment displayed 2 to 5 mismatches with the *Bison bonasus*, *Bison priscus*, and *Bos primigenius* mitochondrial reference sequences. Analysis of the Siréjol bone fragment with the same Bovinae primers yielded a sequence identical to the one obtained from the coprolite. These PCR studies, and subsequent quantitative PCR analyses (Additional file 1: Supplementary methods and Figure S1) also indicated far higher amounts of bovine mitochondrial DNA in the coprolite than in the bone sample. We therefore decided to perform high-throughput sequencing of the coprolite DNA extract and to undertake a PCR approach for analyzing the bone sample.

A total of 601,509,879 Illumina single-pass reads, each at least 20 nucleotides in length, were produced from the coprolite DNA library. The reads were aligned simultaneously to a set of 49 mitochondrial genomes, including the reference mitochondrial genomes for the extinct cave hyena and several species likely to be part of a carnivorous diet. Only the reads matching perfectly, without indel or mismatch, to a unique genome were considered. As shown in Additional file 1: Table S3, this analysis confirmed that the extinct cave hyena was indeed the producer of the coprolite, since the number of reads (7,550) mapping specifically to the cave hyena genome was by far the largest. A large number of reads (3,220) mapped to the European bison (*Bison bonasus*) genome. The number of reads mapping specifically to other genomes then plummeted: 432 reads for *Bos primigenius*, 381 reads for *Bison priscus*, 127 reads for the chamois (*Rupicapra rupicapra*), 60 reads for the musk ox (*Ovibos moschatus*) and less than 54 reads for the other genomes tested. Of importance, only one read mapped specifically to the reference mitochondrial genome of *Homo sapiens*, attesting to the absence of contamination of the library by human DNA. These results thus indicated that the coprolite producer was a cave hyena that had ingested a bovine specimen whose closest known relative is *Bison bonasus*.

### Assembling the bovine mitochondrial genome

Initial analyses had revealed that the coprolite had been produced by a cave hyena that preyed on a bovine related to *Bison bonasus*, hereafter referred to as "the GAO Bovinae". We set out to reconstitute the mitochondrial genomes of both specimens by aligning the Illumina reads to the reference mitochondrial genomes of *Bison bonasus* and *Crocuta crocuta*. The reconstruction of the two genomes was carried out in parallel so as to eliminate spuriously aligned reads (see Methods).

We assembled a 16,325-bp bovine mitochondrial genome, termed GAOseq_Bovinae, from 19,830 unique Illumina reads complemented with 84 consensus PCR sequences. We obtained for GAOseq_Bovinae a 32-fold median coverage, taking into account both the Illumina reads and the consensus PCR sequences. As shown in Additional file 1: Figure S2, coverage by Illumina reads was quite variable along the genome and strongly correlated with the sequence GC content, as already observed in other cases for Illumina sequencing [36]. The length distribution of the 19,830 unique Illumina reads is shown in Additional file 1: Figure S3a. The median read length was 48 base pairs, as expected for ancient, highly fragmented DNA. We analyzed the differences between the Illumina reads and the consensus sequence GAOseq_Bovinae. Only 8,308 mismatches out of 964,592 aligned bases were observed between the 19,830 unique Illumina reads and GAOseq_Bovinae, which corresponds to 99.1% identity. Analysis of the positions of these differences with respect to the read ends showed that the G-to-A substitution rate increases at 3’ ends (Additional file 1: Figure S3b), in agreement with what has already been reported for libraries of ancient DNA constructed with the same procedure [12, 37]. An increased substitution rate at the read ends is considered as a hallmark for authenticating sequences generated from ancient DNA fragments. Such fragments exhibit inflated cytosine deamination rates at 5'-overhangs [38], responsible for G to A transitions at the 3’ end of the opposite, repaired DNA strand.

GAOseq_Bovinae is a complete circular genome that contains the expected number of genes (rRNAs, tRNAs, protein-coding genes) for a mammalian mitochondrial genome, with full-length coding sequences (without internal stop codons) for all 13 protein-coding genes (Additional file 1: Table S5). With respect to the reference mitochondrial genome of *Bison bonasus*, GAOseq_Bovinae exhibits 346 differences, including 5 indels and 341 substitutions with a large majority (96%) of transitions. In comparison, the three available mitochondrial genomes of *Bison bonasus* display less than 55 differences between each other.

**Fig. 3:**
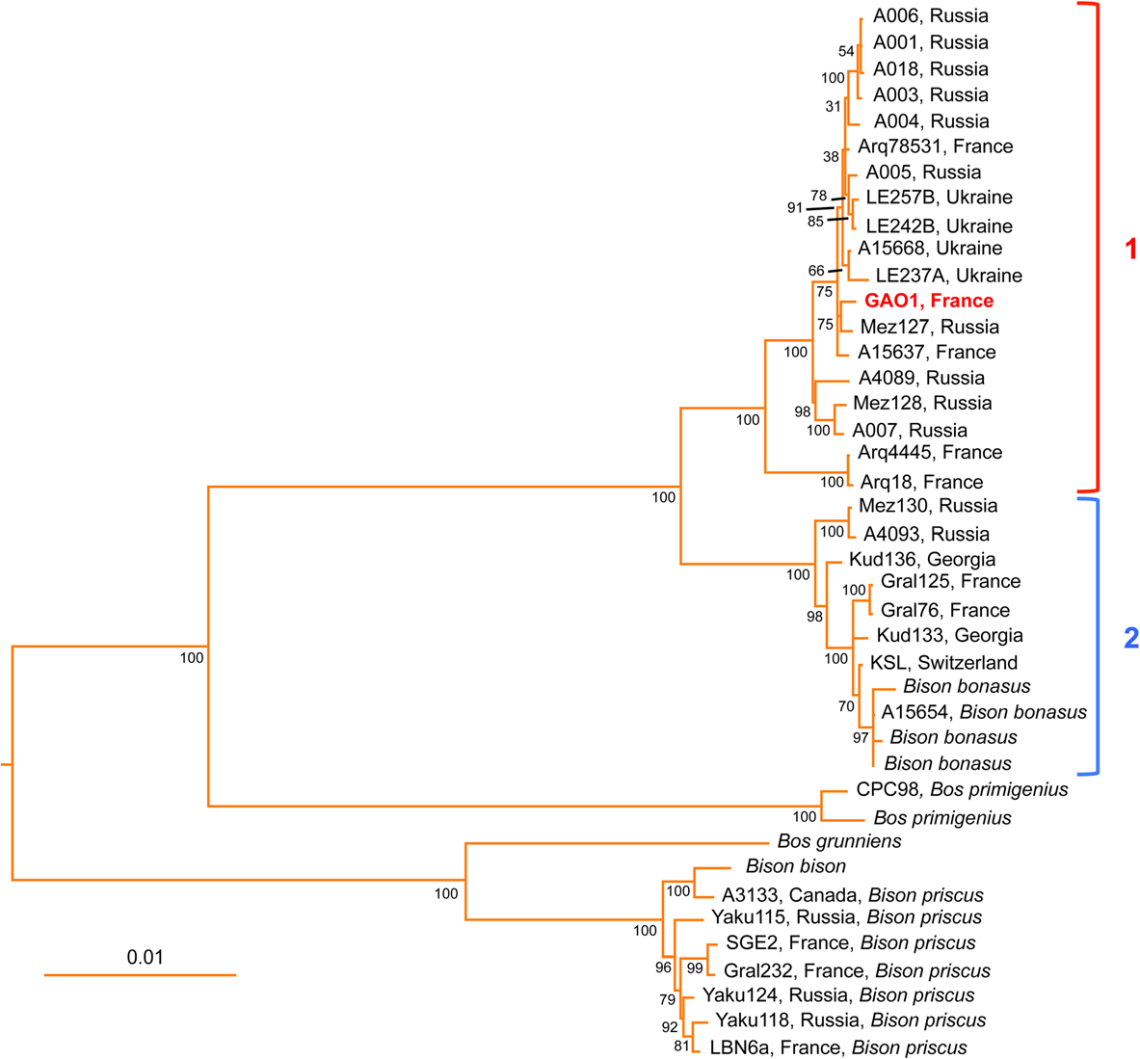
Maximum Likelihood phylogenetic tree of complete mitochondrial genomes of *Bison* and *Bos* species. The tree with the highest log-likelihood is shown drawn to scale, with branch lengths established from the numbers of substitutions per site. The percentages of trees in which the associated taxa clustered together are displayed next to the branches (the bootstrap values were determined with 500 replicates). The GenBank accession numbers of the sequences are listed in Additional file 1: Table S6. Specimen name and country of origin are indicated for ancient and historical samples. The tree was rooted using the sequence of *Bubalus bubalis*. Refer to text for explanation about clade 1 and clade 2

While our manuscript was under review, two articles were published [28, 39] that describe several mitochondrial genomes from Late Pleistocene/Holocene bison specimens related to *Bison bonasus*. We therefore performed a phylogenetic analysis including all these ancient mitochondrial genomes along with the mitochondrial genomes of *Bison bonasus* specimens. We also considered *Bison priscus* specimens from these studies and a previous report from our laboratory [12], as well as the reference mitochondrial genomes of *Bos primigenius*, *Bison bison*, *Bos grunniens* (yak) and *Bubalus bubalis* (swamp buffalo) (Additional file 1: Table S6). Phylogenetic trees were constructed from this dataset with the Maximum Likelihood (ML), Minimum Evolution and Neighbor-Joining methods that all yielded similar topologies. As shown in Fig. 3 for the ML method, GAOseq_Bovinae positions in a well-supported clade (clade 1) that includes all the ancient genomes (dated from 14.3 to more than 50 kya calBP) forming CladeX in [28] and clade Bb1 in [39]. No species from the fossil record has been proposed up to now for this clade which delineates a sister species for *Bison bonasus* ancient (11.7 to more than 50 kya calBP), historical, and extant specimens from clade 2 (corresponding to clade Bb2 in [39]). Mitochondrial genomes of clades 1 and 2 are phylogenetically closer to extant and ancient *Bos primigenius* than to *Bison priscus* and extant American bison.

### Analysis of mitochondrial DNA sequences derived from a *Bison schoetensacki* bone sample

**Fig. 4:**
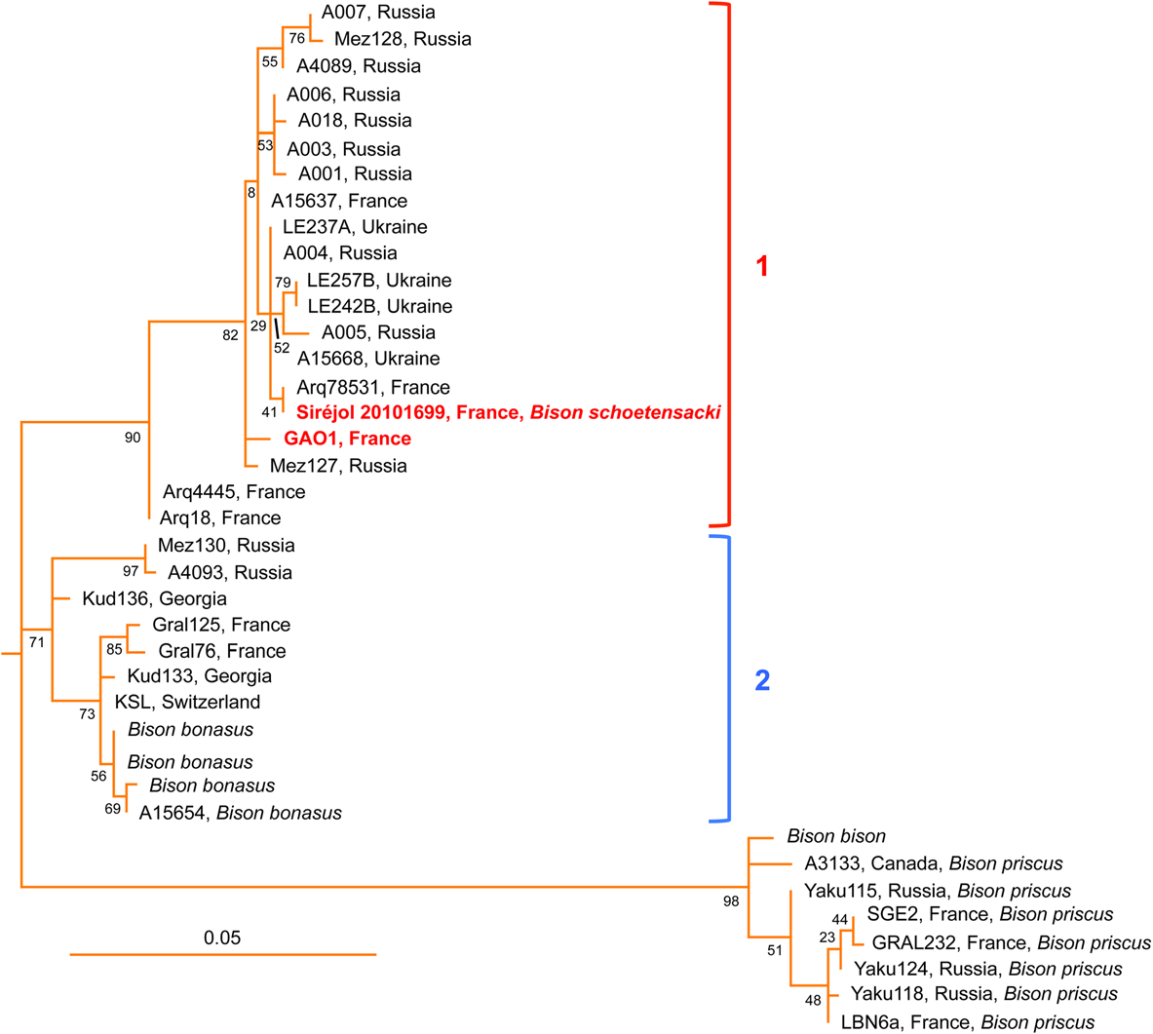
Phylogenetic position of the *Bison schoetensacki* mitochondrial DNA sequence. Maximum Likelihood phylogenetic analysis was performed using the concatenated sequence fragments (609 nucleotides) of the *Bison schoetensacki* Siréjol sample and the orthologous sequences of the indicated bison mitochondrial genomes. The tree with the highest log-likelihood is shown drawn to scale, with branch lengths established from the numbers of substitutions per site. The percentages of trees in which the associated taxa clustered together are displayed next to the branches (the bootstrap values were determined with 500 replicates). The tree was rooted using the sequence of *Bubalus bubalis*. See legend to Fig. 3 for additional details

We explored the phylogenetic relationship between clade 1 and *Bison schoetensacki* sequences by analyzing mitochondrial DNA fragments of the Siréjol bone sample. For this we designed a set of 16 primer pairs allowing PCR amplification of short mitochondrial DNA fragments (Additional file 1: Table S2) that were analyzed through the sequencing of cloned amplicons from several PCR replicates. Concatenation of the consensus sequences of the 16 PCR fragments yielded information on 609 bp of the mitochondrial genome. The phylogenetic tree obtained using this concatenated sequence and orthologous fragments of bison mitochondrial genomes (Fig. 4) strongly supports the position of the Siréjol *Bison schoetensacki* specimen within clade 1 and illustrates the similarity between the *Bison schoetensacki* sequence and the sequences of Arq78531 and GAOseq_Bovinae, from which it differs at 1 and 6 positions, respectively. By contrast, the *Bison schoetensacki* sequence displays 35 to 52 differences with the corresponding fragments of the mitochondrial genomes belonging to clade 2, including the modern *Bison bonasus* sequences, and 64 and 69 differences with the reference mitochondrial genomes of *Bison priscus* and *Bison bison*. These observations support the conclusion that the bovine DNA present in the coprolite originates from a *Bison schoetensacki* specimen and strongly suggest that the clade 1 itself (referred to as CladeX in [28] and as clade Bb1 in [39]) corresponds to the *Bison schoetensacki* species.

### Analysis of *Bison schoetensacki* nuclear sequences

**Fig. 5:**
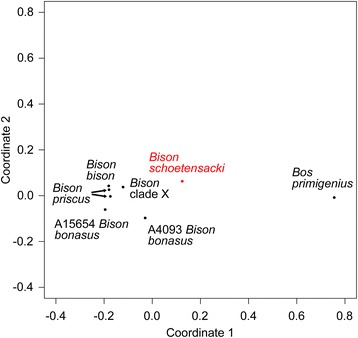
Multidimensional scaling analysis of bovine nuclear SNPs. We used the SNPs genotyped herein in the *Bison schoetensacki* sequences of the coprolite, in a modern *Bison bison* specimen and in the reference genome of *Bos primigenius* (taurine cattle reference UMD 3.1) as well as the SNPs genotyped by Soubrier et al. [28] in the CladeX A006 specimen, in the historical *Bison bonasus* A15654 specimen, in the ancient *Bison bonasus* A4093 specimen, and in the two *Bison priscus* A875 and A3133 specimens

Soubrier et al. [28] analyzed a set of ~ 10,000 genome-wide bovine SNPs from several specimens of their *Bison bonasus*-related CladeX (only one of which, A006, yielded a sufficient amount of information for further analysis), from two *Bison bonasus* specimens (a historical one killed in 1911 and an ancient one, dated >55 kyr) and from two *Bison priscus* specimens (dated 30 and >50 kyr) [28]. In order to compare the bison nuclear sequences of the coprolite to these data, the library reads were mapped against the taurine cattle reference genome and the genotypes of the bovine SNPs were determined. The genotypes of the SNPs for the nuclear genome of a *Bison bison* specimen were also extracted from published genomic data (see Methods). Analysis by multidimensional scaling (Fig. 5) shows that the closest specimen to *Bison shoetensacki* is the CladeX specimen A006 [28]. This result corroborates the conclusions drawn from the mitochondrial sequence data that position the bison sequences of the coprolite in the CladeX/Bb1 sister group to *Bison bonasus*.

## Conclusions

The most ancient specimens of *Bison schoetensacki* in the fossil record date back to the beginning of the Middle Pleistocene (i.e. some 750,000 years ago), but the continuing presence of this species up to the Upper Pleistocene has been a matter of controversy. Our studies of a Siréjol bone fragment provide strong support to the notion that this species existed for a long period of time, extending to the Upper Pleistocene. Moreover, comparison of our mitochondrial and nuclear genome data with those of recent studies suggest that 15,000 to > 50,000-year-old *Bison schoetensacki* remains are present in a number of Eurasian sites. The DNA data show that *Bison schoetensacki* positions in a bison clade referred to as CladeX [28] or Bb1 [39] which should be renamed accordingly. Scenarios of bison evolution considered that *Bison bonasus* evolved either from *Bison priscus* or from *Bison schoetensacki* lineages. The genetic data clearly support the latter rather than the former hypothesis. The divergence date between *Bison bonasus* and *Bison schoetensacki* is nevertheless still unclear since it has been estimated around 120 (152-92) kya and around 246 (283-212) kya in the two studies with different datasets [28, 39]. Opposite conclusions have also been reached regarding the evolution of *Bison bonasus* as due either to incomplete lineage sorting [39] or to hybridization between *Bison priscus* and ancestors of *Bos primigenius* [28]. Progress toward a precise assessment of the timing of Bovinae evolution and of the respective contribution of incomplete lineage sorting and introgression into the emergence of new species calls for the analysis of specimens much older than those studied up to now. Ancient DNA analysis was long considered to be only feasible for Upper Pleistocene specimens, but studies carried out on Middle Pleistocene remains retrieved from permafrost [40] or even sites from temperate climate [41] indicate that genome data for more than 300 kya-old Bovinae specimens are expected in the near future.

## Additional file

Additional file 1: Supplementary text. Supplementary methods, supplementary results, and supplementary references. **Figure S1.** Real-time PCR analysis of the Grotte-aux-Ours coprolite and the Siréjol bone fragment for mitochondrial DNA. PCR was carried out using a TaqMan assay designed using the Grotte-aux-Ours bovine mitochondrial genome sequence (GAOseq_Bovinae). **Figure S2.** Correlation between the coverage by Illumina reads and the GC content of the GAOseq_Bovinae sequence. (a) Coverage for each position of the GAOseq_Bovinae sequence by the 19,830 unique Illumina reads. (b) The GC content of GAOseq_Bovinae was calculated at each base pair (bp) using a 100 bp sliding window. (c) The normalized coverage by Illumina reads and the normalized GC content are shown on the same graph. **Figure S3.** Characteristics of the Illumina reads used to assemble the mitochondrial genome GAOseq_Bovinae. (a) Length distribution of the 19,830 unique Illumina reads. (b) Positions of the mismatches between the 19,830 unique Illumina reads and the GAOseq_Bovinae sequence. **Figure S4.** Maximum Likelihood phylogenetic tree of the mitochondrial genomes of *Crocuta crocuta*. **Table S1.** PCR primers used to amplify bovine mitochondrial DNA fragments from the Grotte-aux-Ours coprolite. **Table S2.** PCR primers used to amplify *Bison schoetensacki* mitochondrial DNA fragments from the Siréjol bone sample. **Table S3.** The set of mitochondrial genomes against which the Illumina reads were aligned to determine the identity of the coprolite producer and of its prey. **Table S4.** AMS radiocarbon and stable isotope data for *Bison schoetensaki* specimen 20101699. **Table S5.** Annotation of the *Bison schoetensacki* mitochondrial genome sequence. **Table S6.** The set of mitochondrial genomes used for phylogenetic analyses. (PDF 1291 kb)
