# Supplementary material for: Genome data on the extinct Bison schoetensacki establish it as a sister species of the extant European bison (Bison bonasus)
Source: BMC Evol Biol. 2017 Feb 10;17:48. doi: 10.1186/s12862-017-0894-2 (PMC5303235; doi:10.1186/s12862-017-0894-2)
Supplement: Additional file 1: — Supplementary text. Supplementary methods, supplementary results, and supplementary references. Figure S1. Real-time PCR analysis of the Grotte-aux-Ours coprolite and the Siréjol bone fragment for mitochondrial DNA. PCR was carried out using a TaqMan assay designed using the Grotte-aux-Ours bovine mitochondrial genome sequence (GAOseq_Bovinae). Figure S2. Correlation between the coverage by Illumina reads and the GC content of the GAOseq_Bovinae sequence. (a) Coverage for each position of the GAOseq_Bovinae sequence by the 19,830 unique Illumina reads. (b) The GC content of GAOseq_Bovinae was calculated at each base pair (bp) using a 100 bp sliding window. (c) The normalized coverage by Illumina reads and the normalized GC content are shown on the same graph. Figure S3. Characteristics of the Illumina reads used to assemble the mitochondrial genome GAOseq_Bovinae. (a) Length distribution of the 19,830 unique Illumina reads. (b) Positions of the mismatches between the 19,830 unique Illumina reads and the GAOseq_Bovinae sequence. Figure S4. Maximum Likelihood phylogenetic tree of the mitochondrial genomes of Crocuta crocuta. Table S1. PCR primers used to amplify bovine mitochondrial DNA fragments from the Grotte-aux-Ours coprolite. Table S2. PCR primers used to amplify Bison schoetensacki mitochondrial DNA fragments from the Siréjol bone sample. Table S3. The set of mitochondrial genomes against which the Illumina reads were aligned to determine the identity of the coprolite producer and of its prey. Table S4. AMS radiocarbon and stable isotope data for Bison schoetensaki specimen 20101699. Table S5. Annotation of the Bison schoetensacki mitochondrial genome sequence. Table S6. The set of mitochondrial genomes used for phylogenetic analyses. (PDF 1291 kb) [file 12862_2017_894_MOESM1_ESM.pdf]

Supplementary methods

Real-time PCR

To set up a real-time PCR assay, we used the custom TaqMan assay design tool available on the Thermo Fisher Scientific website (https://www.thermofisher.com/order/custom-genomic-products/tools/gene-expression/). The TaqMan assay targets a 75-bp fragment of the cytochrome B sequence from the complete bovine mitochondrial genome sequence (GAOseq_Bovinae) reconstructed from the Grotte-aux-Ours sample. The sequence of the forward (F) and reverse primer (R) and of the FAM-labeled TaqMan-MGB probe (P) are as follows: F, 5’- CTACTAGTACTATTCGCACCCGA -3’; R, 5’- AGGCGTGTTAAGTGGATTTGCT -3’; P, 5’- CCTCCTCGGAGACCCAG -3’.

Real-time PCR was carried out in a 20-µl reaction volume containing 10 µl of 2X TaqMan universal PCR Master Mix, 1 µl of the 20X probe-primers mixture, and 0.3 µl of mock or DNA extracts or an equivalent volume of water (blank samples). Amplification was performed in a CFX96 touch real-time PCR detection system (BioRad, Hercules, CA, USA) and included an initial step for enzyme activation (95°C, 10 min) followed by 45 PCR cycles (95°C, 15 s; 60°C, 1 min). Only DNA extracts yielded amplification signals. Data were analyzed by calculating the cycle threshold (C_T), which corresponds to the number of PCR cycles required for the fluorescent signal to exceed the background level.

Metagenomic analyses

A total of one million reads randomly sampled from the four sequencing lanes was analyzed by BLAST [1] against the GenBank *nr/nt* database with the following options: *-task* megablast *-word_size* 19 *-max_target_seqs* 1 *-gapopen* 5 *-gapextend* 2 *-evalue* 0.01 -

culling_limit 1. Only the hits that display an E-value lower than 0.01 were considered significant.

Analysis of ancient DNA damage

The mismatches between the 19,830 unique Illumina reads finally selected and the GAOseq_Bovinae sequence were analyzed using in-house Python and R scripts available upon request. Briefly, the SAM files generated by BWA were parsed to retrieve the aligned reads and the corresponding genomic regions, which were compared for mismatches.

Phylogenetic analysis of the cave hyena sequence

The phylogenetic relationships between GAOseq_Crocuta and the three complete mitochondrial genomes of *Crocuta crocuta* available on the NCBI website (http://www.ncbi.nlm.nih.gov/, last accessed February 23, 2016) were inferred using the ML, ME and NJ methods. The reference mitochondrial genome of the striped hyena (*Hyaena hyaena*, GenBank accession number NC_020669.1) was taken as an outgroup. We proceeded as described in [2] and discarded for the phylogenetic analysis the domains of the control region that display taxon-related insertions or deletions, including the two portions with tandem repeats (positions 212 to 535, and 16258 to 16832, according to the reference mitochondrial genome of *Crocuta crocuta*). The ML method was based on the Hasegawa-Kishino-Yano model. The analysis involved a total of 16,099 positions.

Supplementary results

Assembly of the cave hyena mitochondrial genome

We assembled for the hyena specimen that produced the coprolite an almost complete mitochondrial genome (16,122 bp), called GAOseq_Crocuta. We obtained a median coverage of 27 without taking into account the domains of the control region that display tandem repeats (see Supplementary Methods), and 119 positions that were covered by less than two reads. The read length distribution, with a median value of 49 bp, and the increasing number of differences at the 3' ends of the Illumina reads compared to GAOseq_Crocuta were similar to those observed for the reads mapping to GAOseq_Bovinae and therefore mark these reads as corresponding to ancient DNA templates.

The 16,122-bp GAOseq_Crocuta sequence exhibits only 23 differences with the *Crocuta crocuta* cave hyena reference mitochondrial genome. To determine more precisely its phylogenetic position, we compared GAOseq_Crocuta with the two mitochondrial genomes available for the extinct cave hyena and with the mitochondrial genome of a modern spotted hyena, taking as an outgroup the striped hyena (*Hyaena hyaena*) genome. It has to be noted that the extinct Eurasian cave hyena and the extant African spotted hyena correspond to the same species [2,3], namely *Crocuta crocuta*, even if we use the two vernacular names for the sake of clarity. Phylogenetic trees were constructed from this dataset with the ML, ME and NJ methods. As shown in Additional file 1: Figure S4, for the ML method, GAOseq_Crocuta and the two cave hyena mitochondrial genomes form a well-supported clade (100% bootstrap support, 1,000 replicates). Similar results were obtained with the ME and NJ methods (data not shown), which confirms the proximity of GAOseq_Crocuta with Pleistocene cave hyena genomes.

Metagenome analysis

To gain some complementary insight into the content of the Illumina library, one million randomly selected reads were aligned by BLAST against the GenBank *nt/nr* database,

which yielded 212,802 significant hits. The majority of these hits (73.5%) corresponded to Bacteria but Eukaryota represented 25.6% of the total. In the bacterial metagenome, the predominant classes were Actinobacteria (65.5% of bacterial hits), Betaproteobacteria (6.5%), Alphaproteobacteria (5.7%) and Gammaproteobacteria (4.7%). Similar distributions of microbial diversity have been described for ancient DNA extracts (e.g. [4]). Among the Eukaryota, the classes most represented were the Mammalia (76% of eukaryotic hits) and the Chromadorea (a class of the Nematoda, 10.4% of eukaryotic hits).

Supplementary references

1. Camacho C, Coulouris G, Avagyan V, Ma N, Papadopoulos J, Bealer K, et al. BLAST+: architecture and applications. BMC Bioinformatics. 2009;10:421.

2. Bon C, Berthonaud V, Maksud F, Labadie K, Poulain J, Artiguenave F, et al. Coprolites as a source of information on the genome and diet of the cave hyena. Proc Biol Sci 2012;279:2825–30.

3. Stuart AJ, Lister AM. New radiocarbon evidence on the extirpation of the spotted hyaena (*Crocuta crocuta* (Erxl.)) in northern Eurasia. Quat Sci Rev. 2014;96:108–16.

4. Sarkissian Der C, Ermini L, Jónsson H, Alekseev AN, Crubezy E, Shapiro B, et al. Shotgun microbial profiling of fossil remains. Mol Ecol. 2014;23:1780–98.

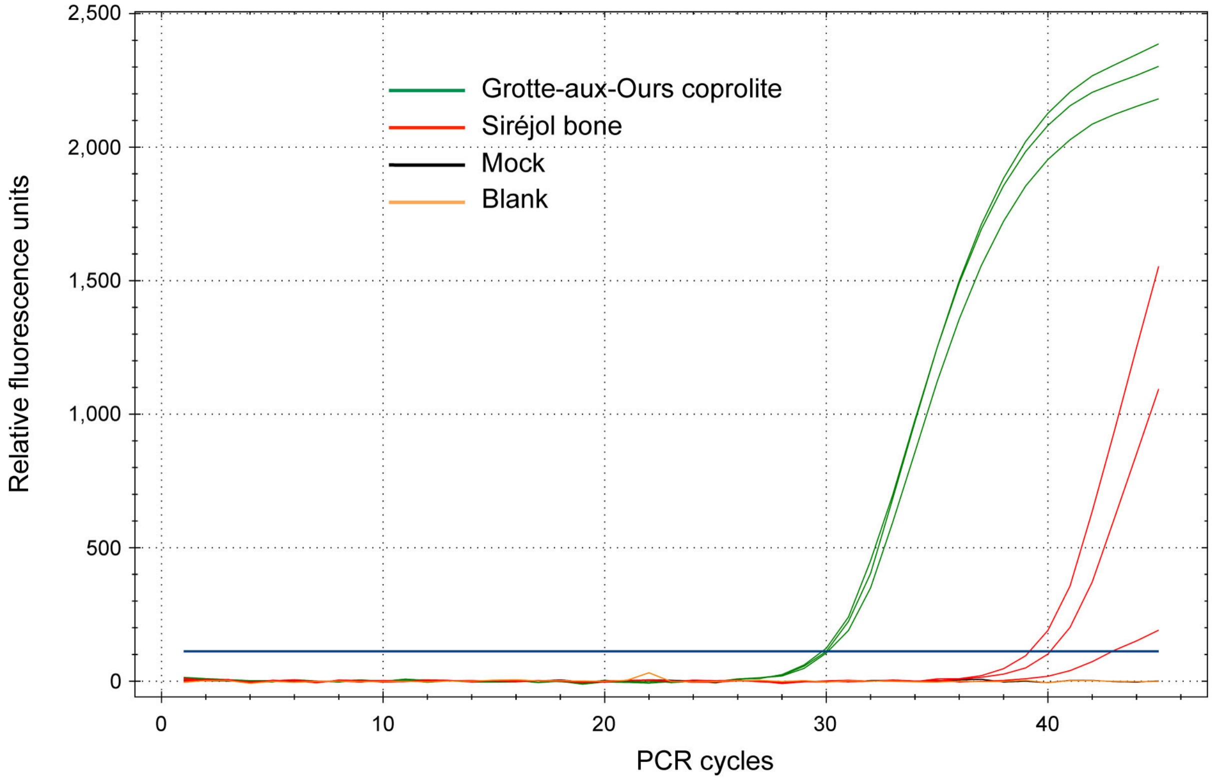

Figure S1. Real-time PCR analysis of the Grotte-aux-Ours coprolite and the Siréjol bone fragment for mitochondrial DNA. PCR was carried out using a TaqMan assay designed using the Grotte-aux-Ours bovine mitochondrial genome sequence (GAOseq_Bovinae). The fragment detected corresponds to part of the cytochrome B gene. The figure displays results obtained for triplicate aliquots corresponding to 0.3% of each DNA extract. The horizontal blue line indicates the threshold used for calculating C_T values. Negative controls (mock extract and PCR blank) failed to yield amplification signals.

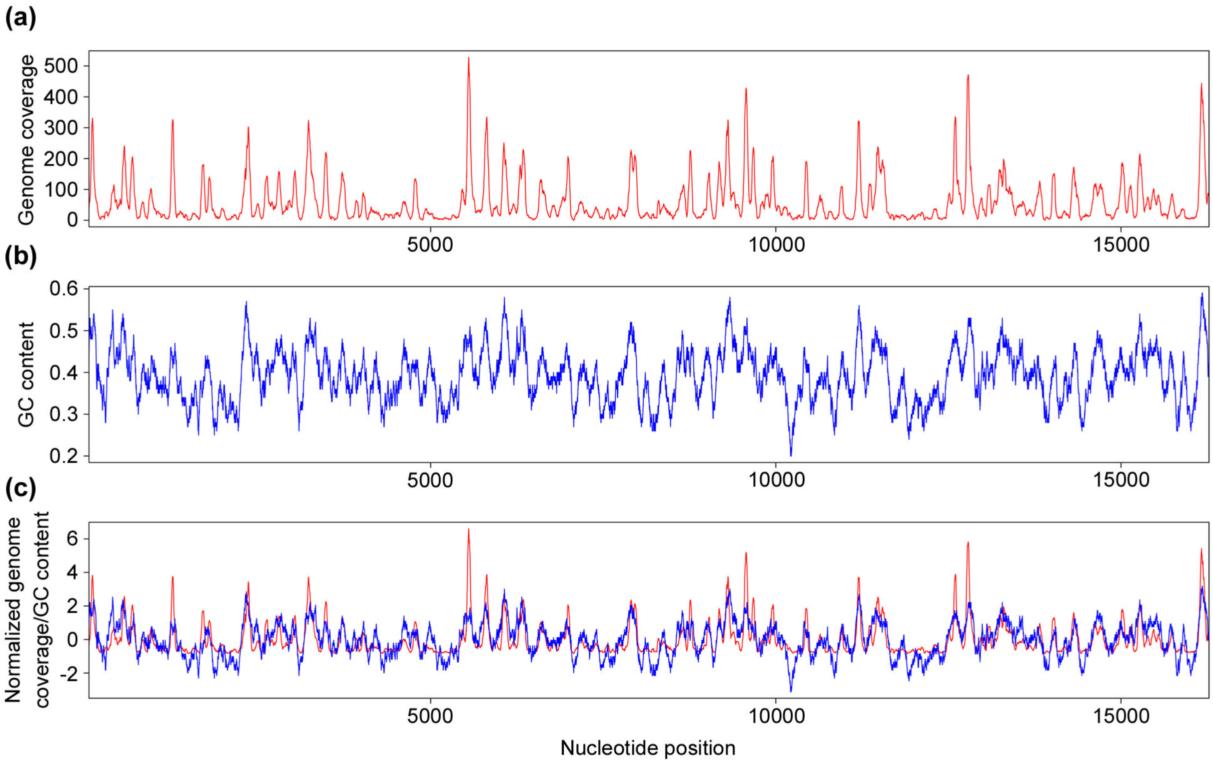

Figure S2. Correlation between the coverage by Illumina reads and the GC content of the GAOseq_Bovinae sequence. (a) Coverage for each position of the GAOseq_Bovinae sequence by the 19,830 unique Illumina reads. (b) The GC content of GAOseq_Bovinae was calculated at each base pair (bp) using a 100 bp sliding window. (c) The normalized coverage by Illumina reads and the normalized GC content are shown on the same graph. The two variables are highly correlated (Spearman's correlation coefficient r = 0.79, $P = 10^{-35}$).

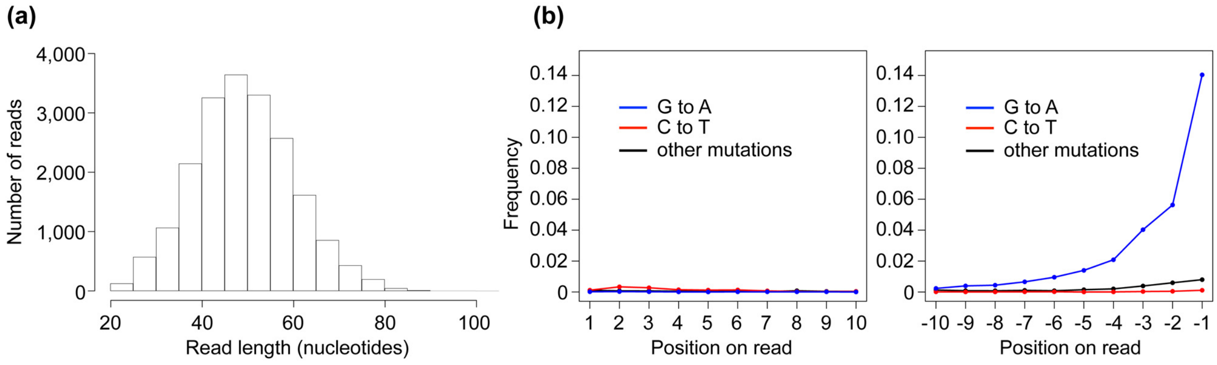

Figure S3. Characteristics of the Illumina reads used to assemble the mitochondrial genome GAOseq_Bovinae. (a) Length distribution of the 19,830 unique Illumina reads. (b) Positions of the mismatches between the 19,830 unique Illumina reads and the GAOseq_Bovinae sequence. The frequencies of the 12 possible mismatches are plotted as a function of the distance from the 5' end (left part) or the 3' end (right part) of reads. Since the Illumina reads are at least 20 nucleotides in length, only the ten 5' and ten 3' most positions are shown.

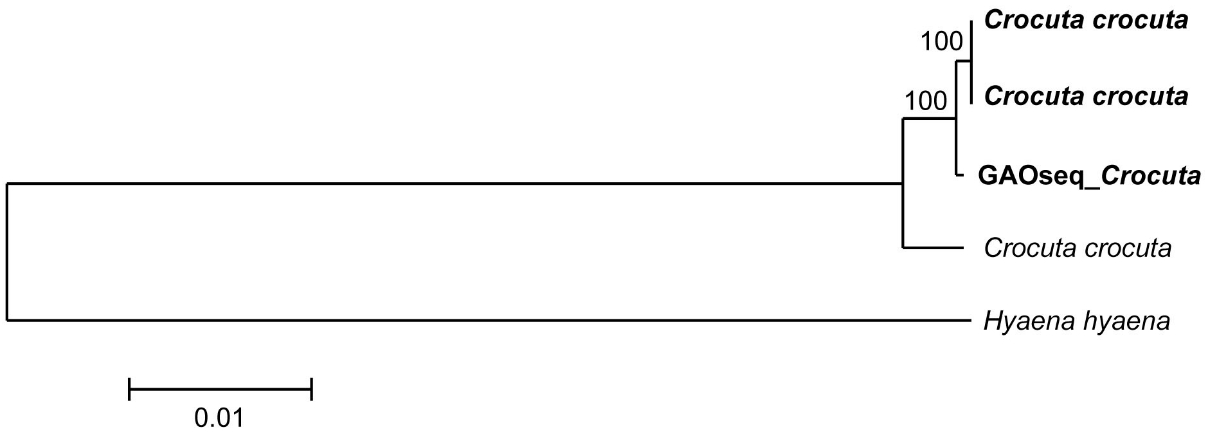

Figure S4. Maximum Likelihood phylogenetic tree of the mitochondrial genomes of *Crocuta crocuta*. The tree with the highest log-likelihood is shown drawn to scale, with branch lengths established from the numbers of substitutions per site. The percentages of trees in which the associated taxa clustered together are displayed next to the branches (the bootstrap values were determined from 1,000 replicates). The GenBank accession numbers for the *Crocuta crocuta* genomes are as following (from top to bottom): JF894379.1, NC_020670.1 and JF894377.1. The names in bold correspond to ancient DNA sequences.

Table S1. PCR primers used to amplify bovine mitochondrial DNA fragments from the Grotte-aux-Ours coprolite.

Primer pair	Primer position, sequence	Amplicon (bp)
1	170F, 5'-TGGACATTACAGTCAATGGT-3'<br>283R, 5'-AAGCAACTAGGGAAAAGTCTG-3'	114
2	1419F, 5'-CCTAGAAGACTTCATTCAT-3'<br>1538R, 5'-CTCCTATACTTTAAATTGGGAA-3'	120
3	1547F, 5'-CTAAGTACGGCGCTATAGAGA-3'<br>1634R, 5'-GTACAAGGGGTAATCTTTGCT-3'	88
4	1992F, 5'-GCGTTAAAGCTCAACAACA-3'<br>2080R, 5'-ATTCTATAATAGATTAGTCCAGT-3'	89
5	2154F, 5'-ATTCTGACCACTAACAGCTA-3'<br>2235R, 5'-TGCACTCCTGTGTTGGATT-3'	82
6	3064F, 5'-TTTACATCCAGAGATTCAAATCC-3'<br>3156R, 5'-GAATGCTACGGCCAATAGGA-3'	93
7	3564F, 5'-AATAAGTGGATCCTTTACCCT-3'<br>3648R, 5'-TATTGCTAGAGGTCATGCTGG-3'	85
8	4040F, 5'-TCCCGCCACTGACATAAGAA-3'<br>4129R, 5'-AGTTCTAGAAATAAGAGGGTT-3'	90
9	4250F, 5'-ACCCTTCCCGTACTAATAAACC-3'<br>4360R, 5'-TCCGATTCAGACAAGTAGTCAG-3'	111
10	4831F, 5'-AATAACAGCAGTACTACCATA-3'<br>4935R, 5'-GTGGAATTGGCCATGAAT-3'	105
11	5032F, 5'-CCTATCTGGGTTCATACCAA-3'<br>5134R, 5'-TAGGTTTAGTAGAGCTGTAATTGC-3'	103
12	5083F, 5'-CAACAGCATCATTCTACC-3'<br>5162R, 5'-TAGAGTATGTGAGTCGTA-3'	80
13	5201F, 5'-ATAAAATGACAATTTCCCCTT-3'<br>5297R, 5'-CTGATAGTATTGGTGTGAGT-3'	97
14	6432F, 5'-TGGATTTGGAATAATCTCCCATA-3'<br>6531R, 5'-AAATCCGATTGACATTATAGCC-3'	100
15	7039F, 5'-ACTATCTCATCAATAGGCTCAT-3'<br>7130R, 5'-ACTTCTCGTTTAGATGCAAA-3'	92
16	7411F, 5'-ATCACCAATCATAGAGGAACT-3'<br>7485R, 5'-ACTAATGAGCTAATTAAGAAGACA-3'	75
17	7571F, 5'-ATTTTGCCCGCTATTATCTT-3'<br>7687L, 5'-GTATCATTGATGTCCTATGGTT-3'	117
18	11044F, 5'-TCCCTAAACTTCCTAATACTCCA-3'<br>11133R, 5'-AGCTATTATACATGCTAGTCA-3'	90
19	11682F, 5'-CTTTTCATGATCTAACATTACAAT-3'<br>11769R, 5'-AGTTATAATTAGCATGTATAGGGA-3'	88
20	11876F, 5'-ACCCAAAAATTATTCTAGGACCTC-3'<br>11977R, 5'-TCGGTAAATAAGAAGGTAATGAGTT-3'	102
21	12110F, 5'-AACATATTCGCCTCATTCTC-3'<br>12202R, 5'-GTAATTGGAAGATTTGTAGGTGT-3'	93
22	12337F, 5'-CCTCAGCTTTAAAATAGACTACTTC-3'<br>12437R, 5'-CTGAGTGTATGTATCATATTGAGA-3'	101

23	13847F, 5’-AAAGGCCTAATCAAACTATACTTC-3’<br>13941R, 5’-GGTTATTATAGAAATTACTCGTGGA-3’	95
24	14067F, 5’-ATCTCCCAAACCATTAAACTCA-3’<br>14165R, 5’-GCATTTGTTACTGGCTTGTTGA-3’	99
25	14380F, 5’-AACCCTACAAAACCTATCACA-3’<br>14472R, 5’-ATCATTAGTCATGGTTAGATTCC-3’	93
26	15910F, 5’-GCACACACCCCATACACA-3’<br>16019R, 5’-GGGGCATATAATTTAATGTACT-3’	110
27	16012F, 5’-TATGCCCCATGCGTATAAGCAA-3’<br>16149R, 5’-TCAAGCTCGCGATCTAATGGA-3’	138

Primer sequences were designed using the provisional bovine mitochondrial genome sequence BB2seq. The position of each forward (F) and reverse (R) primer is numbered according to the final GAOseq_Bovinae sequence.

Table S2. PCR primers used to amplify *Bison schoetensacki* mitochondrial DNA fragments from the Siréjol bone sample.

Primer pair	Primer position, sequence	Amplicon (bp)
1	1451F, 5’-AACTAAACCTAGCCCAAA -3’<br>1525R, 5’-AATTGGGAATAAATGTTTTG-3’	75
2	1547F, 5’-CTAAGTACGGCGCTATAGAGA-3’<br>1634R, 5’-GTACAAGGGGTAATCTTTGCT-3’	88
3	1992F, 5’-GCGTTAAAGCTCAACAACA-3’<br>2080R, 5’-ATTCTATAATAGATTAGTCCAGT-3’	89
4	2154F, 5’-ATTCTGACCACTAACAGCTA-3’<br>2235R, 5’-TGCACTCCTGTGTTGGATT-3’	82
5	5083F, 5’-CAACAGCATCATTCTACC-3’<br>5162R, 5’-TAGAGTATGTGAGTCGTA-3’	80
6	7039F, 5’-ACTATCTCATCAATAGGCTCAT-3’<br>7130R, 5’-ACTTCTCGTTTAGATGCAAA-3’	92
7	7411F, 5’-ATCACCAATCATAGAGGAACT-3’<br>7485R, 5’-ACTAATGAGCTAATTAAGAAGACA-3’	75
8	9831F, 5’-ATACTAGCCCTCCTGACCAA-3’<br>9890R, 5’-TCAGAATGCGATGATGACAAG-3’	60
9	11044F, 5’-TCCCTAAACTTCCTAATACTCCA-3’<br>11133R, 5’-AGCTATTATACATGCTAGTCA-3’	90
10	15033F, 5’-CCTTACCCGATTTTTCGC-3’<br>15104R, 5’-AGTAGATGAACTATGGCAAT-3’	72
11	15103F, 5’-CTATTCCTCCACGAAACAGGT-3’<br>15177R, 5’-GGGGTGGAATGGAATTTTGTCT-3’	75
12	15356F, 5’-GATCAATCCCCAACAAAC-3’<br>15347R, 5’-GTGTGTAGTAGGGGAATTAGA-3’	82
13	16012F, 5’-TATGCCCCATGCGTATAA-3’<br>16076R, 5’-TAGTAATTGTATGTATTATGT-3’	65
14	16090F, 5’-CTTTATGTCAAGCTCATTCTT-3’<br>16176R, 5’-GGTTGCTGGTTTCACGC-3’	87
15	16137F, 5’-ATCGCGAGCTTGATTACC-3’<br>16211R, 5’-GGCCCGGAGCGAGAAGAG-3’	75
16	16169F, 5’-CAGCAACCCGCTAGGCAAA-3’<br>16244R, 5’-AAGTTCATTAAATAGCGACCCC-3’	76

Primers 1, 8, and 10-16 were designed using the final GAOseq_Bovinae sequence. Primers 2-7 and 9, also used to analyze the Grotte-aux-Ours coprolite, were designed using the provisional bovine mitochondrial genome sequence BB2seq. The position of each forward (F) and reverse (R) primer is numbered according to the final GAOseq_Bovinae sequence.

Table S3. The set of mitochondrial genomes against which the Illumina reads were aligned to determine the identity of the coprolite producer and of its prey.

Scientific name	Vernacular name	GenBank number	Number of reads
Crocuta crocuta	Cave hyena	NC_020670	7550
Bison bonasus	European bison	HM045017	3220
Bos primigenius	Aurochs	GU985279	432
Bison priscus	Steppe bison	KM593920	381
Rupicapra rupicapra	Chamois	FJ207539	127
Ovibos moschatus	Musk ox	FJ207536	60
Saiga tatarica	Saiga antelope	JN632700	53
Capreolus capreolus	Roe deer	JN632610	51
Capra pyrenaica	Iberian ibex	FJ207528	48
Capra ibex	Alpine ibex	FJ207526	46
Alces alces	Eurasian elk	JN632595	46
Cervus elaphus	Red deer	AB245427	45
Martes foina	Beech marten	HM106325	40
Rangifer tarandus	Reindeer	AB245426	39
Ursus spelaeus	Cave bear	EU327344	35
Equus przewalskii	Przewalski's horse	JN398403	34
Equus asinus	Ass	X97337	30
Canis lupus	Gray wolf	DQ480505	26
Sus scrofa	Domectic pig	AP003428	23
Equus caballus	Horse	X79547	20
Sorex unguiculatus	Long-clawed shrew	AB061527	16
Coelodonta antiquitatis	Woolly rhinoceros	FJ905813	16
Lepus europaeus	European hare	AJ421471	15
Oryctolagus cuniculus	Rabbit	AJ001588	13
Talpa europae	European mole	Y19192	11
Otis tarda	Great bustard	FJ751803	10
Sciurus vulgaris	Red squirrel	AJ238588	9
Apodemus agrarius	Striped field mouse	JN629047	7
Mammuthus primigenius	Woolly mammoth	DQ316067	6
Rhinolophus ferrumequinum	Greater horseshoe bat	JX084273	6
Oreocryptophis porphyraceus	Black-banded rat snake	GQ181130	5
Gallus gallus	Chicken	X52392	5
Accipiter gentilis	Northern goshawk	AP010797	4
Alectoris chukar	Chukar partridge	FJ752426	3
Falco peregrinus	Peregrine falcon	AF090338	3
Erinaceus europaeus	European hedgehog	X88898	3
Apis mellifera scutellata	African honeybee	KJ601784	2
Buteo buteo	Common buzzard	AF380305	2
Microtus kikuchii	Taiwan vole	AF348082	2
Anas platyrhynchos	Mallard	EU009397	2
Podarcis muralis	Common wall lizard	FJ460597	2

Corvus frugilegus	Rook	Y18522	1
Homo sapiens neanderthalensis	Neandertal	AM948965	1
Homo sapiens	Human	NC_012920	1
Salmo salar	Atlantic salmon	U12143	0
Salmo trutta trutta	Sea trout	AM910409	0
Alytes obstetricans	Common midwife toad	AY585337	0
Anguilla anguilla	European eel	AP007233	0
Esox lucius	Northern pike	AP004103	0

The number of reads matching perfectly and specifically a given genome is indicated in the last column.

Table S4. AMS radiocarbon and stable isotope data for *Bison schoetensaki* specimen 20101699.

Chemical Fraction	^{14}C Age ± 1 SD, RC yr	AMS Lab No	%N	%C	C/N Atomic %	δ^{13}C ‰ VPDB	δ^{15}N ‰ AIR	CAL BP 2 SD (95.4% C.I.)
KOH-Extracted Decalcified Collagen	32,316±215	D-AMS 012204	14.50	44.48	3.07	-21.20	9.08	—
Gelatin	32,623±200	D-AMS 012205	16.41	52.06	3.17	-21.14	8.98	—
Average Date	32,469±147		—	—	—	—	—	36,765-36,001

Table S5. Annotation of the *Bison schoetensacki* mitochondrial genome sequence.

Feature	Strand	Start	Stop	Length
D-loop	+	1	361	361
tRNA-Phe	+	362	428	67
s-rRNA	+	429	1384	956
tRNA-Val	+	1385	1451	67
l-rRNA	+	1452	3022	1571
tRNA-Leu	+	3023	3087	65
ND1	+	3100	4055	956
tRNA-Ile	+	4056	4124	69
tRNA-Gln	-	4122	4193	72
tRNA-Met	+	4196	4264	69
ND2	+	4265	5306	1042
tRNA-Trp	+	5307	5373	67
tRNA-Ala	-	5375	5443	69
tRNA-Asn	-	5445	5517	73
rep_origin	+	5518	5548	31
tRNA-Cys	-	5550	5616	67
tRNA-Tyr	-	5617	5684	68
COX1	+	5686	7230	1545
tRNA-Ser	-	7228	7298	71
tRNA-Asp	+	7303	7371	69
COX2	+	7373	8056	684
tRNA-Lys	+	8060	8126	67
ATP8	+	8128	8328	201
ATP6	+	8289	8969	681
COX3	+	8969	9749	781
tRNA-Gly	+	9753	9821	69
ND3	+	9822	10168	347
tRNA-Arg	+	10169	10237	69
ND4L	+	10238	10534	297
ND4	+	10528	11905	1378
tRNA-His	+	11906	11975	70
tRNA-Ser	+	11976	12035	60
tRNA-Leu	+	12037	12106	70
ND5	+	12107	13927	1821
ND6	-	13911	14438	528
tRNA-Glu	-	14439	14507	69
CYTB	+	14512	15651	1140
tRNA-Thr	+	15656	15724	69
tRNA-Pro	-	15724	15789	66
D-loop	+	15790	16325	536

Table S6. The set of mitochondrial genomes used for phylogenetic analyses

Specimen	Site	Country	GenBank	Species	Age (yr calBP)§	14C ID	Reference
A006	Sur'ya	Russia	KX592187.1	*Bison* (clade X)	22,992-22,490	OxA-14550	10.1038/ncomms13158
A001	Rasik	Russia	KX592185.1	*Bison* (clade X)	15,152-14,510	OxA-14558	10.1038/ncomms13158
A018	Sur'ya	Russia	KX592184.1	*Bison* (clade X)	16,000-15,485	OxA-14552	10.1038/ncomms13158
A003	Voronovka	Russia	KX592186.1	*Bison* (clade X)	15,075-14,321	OxA-14948	10.1038/ncomms13158
A004	Rasik	Russia	KX592188.1	*Bison* (clade X)	23,155-22,586	OxA-14545	10.1038/ncomms13158
Arq78531	Aven Arquet	France	KX898007.1	*Bison* (clade B1)			10.1186/s12915-016-0317-7
A005	Ladeinyi Kamen	Russia	KX592189.1	*Bison* (clade X)	18,750-18,400	OxA-14556	10.1038/ncomms13158
LE257B	Amvrosievka	Ukraine	KX592183.1	*Bison* (clade X)			10.1038/ncomms13158
LE242B	Amvrosievka	Ukraine	KX592179.1	*Bison* (clade X)			10.1038/ncomms13158
A15668	Vinnicki oblast	Ukraine	KX592180.1	*Bison* (clade X)	16,547-16,182	ETH-66330	10.1038/ncomms13158
LE237A	Amvrosievka	Ukraine	KX592177.1	*Bison* (clade X)			10.1038/ncomms13158
Mez127	Mezmaiskaya	Russia	KX898015.1	*Bison* (clade B1)	46,500-44,600		10.1186/s12915-016-0317-7
A15637	Aven Arquet	France	KX592178.1	*Bison* (clade X)	>48,000	OxA-32490	10.1038/ncomms13158
A4089	Mezmaiskaya	Russia	KX592182.1	*Bison* (clade X)	>59,400	OxA-19197	10.1038/ncomms13158
Mez128	Mezmaiskaya	Russia	KX898016.1	*Bison* (clade B1)	47,000-44,000		10.1186/s12915-016-0317-7
A007	Sur'ya	Russia	KX592181.1	*Bison* (clade X)	70,691-53,641	OxA-14548	10.1038/ncomms13158
Arq4445	Aven Arquet	France	KX898006.1	*Bison* (clade B1)			10.1186/s12915-016-0317-7
Arq18	Aven Arquet	France	KX898005.1	*Bison* (clade B1)			10.1186/s12915-016-0317-7
Mez130	Mezmaiskaya	Russia	KX898017.1	*Bison bonasus*	51,000-47,770		10.1186/s12915-016-0317-7
A4093	Mezmaiskaya	Russia	KX592175.1	*Bison bonasus*	>56,300	OxA-19201	10.1038/ncomms13158
Kud136	Kudaro	Georgia	KX898013.1	*Bison bonasus*	38,500-37,000		10.1186/s12915-016-0317-7
GRAL125	Igue-du-Gral	France	KX898009.1	*Bison bonasus*			10.1186/s12915-016-0317-7
GRAL76	Igue-du-Gral	France	KX898008.1	*Bison bonasus*	12,100-11,700		10.1186/s12915-016-0317-7
Kud133	Kudaro	Georgia	KX898012.1	*Bison bonasus*	22,500-22,100		10.1186/s12915-016-0317-7
KSL	Kesslerloch	Switzerland	KX898011.1	*Bison bonasus*	14,300-13,800		10.1186/s12915-016-0317-7
	Wrocław Zoo	Poland	NC_014044.1	*Bison bonasus*			10.1007/s13353-012-0090-4
A15654	Kuban Oblast	Russia	KX592176.1	*Bison bonasus*	¶		10.1038/ncomms13158
			HQ223450	*Bison bonasus*			
			JN632602.1	*Bison bonasus*			10.1016/j.crvi.2011.11.002
CPC98	Carsington Pasture	England	NC_013996.1	*Bos primigenius*	6,200-5,650		10.1371/journal.pone.0009255
		Korea	NC_006853.1	*Bos primigenius* (modern)			
			NC_006380.3	*Bos grunniens*			
		USA	NC_012346.1	*Bison bison*			10.1016/j.cub.2008.01.019
A3133	Irish Gulch	Canada	KX592174.1	*Bison priscus*	31,044-30,092	OxA-22141	10.1038/ncomms13158
Yaku115	Chersky	Russia	KX898018.1	*Bison priscus*			10.1186/s12915-016-0317-7
SGE2	Trois-Frères	France	NC_027233.1	*Bison priscus*	19,390-18,940	UCIAMS-144544	10.1371/journal.pone.0128267
GRAL232	Igue-du-Gral	France	KX898010.1	*Bison priscus*			10.1186/s12915-016-0317-7
Yaku124	Chersky	Russia	KX898020.1	*Bison priscus*			10.1186/s12915-016-0317-7
Yaku118	Chersky	Russia	KX898019.1	*Bison priscus*			10.1186/s12915-016-0317-7
LBN6A	La Berbie	France	KX898014.1	*Bison priscus*			10.1186/s12915-016-0317-7
		China	NC_006295.1	*Bubalus bubalis*			

Specimen names are listed for sequences obtained from ancient and historical samples. §: age is only provided for samples for which a direct radiocarbon determination is available; ¶, historical specimen hunted in 1911. For modern samples, when available in the GenBank record the country of origin of the specimen is indicated. Sequences are displayed according to the order in which they appear, from top to bottom, in Fig. 3.
